# The associations between indices of patellofemoral geometry and knee pain and patella cartilage volume: a cross-sectional study

**DOI:** 10.1186/1471-2474-11-87

**Published:** 2010-05-10

**Authors:** Stephanie K Tanamas, Andrew J Teichtahl, Anita E Wluka, Yuanyuan Wang, Miranda Davies-Tuck, Donna M Urquhart, Graeme Jones, Flavia M Cicuttini

**Affiliations:** 1 School of Public Health and Preventive Medicine, Monash University, Commercial Road, Melbourne, 3004, Australia; 2 Menzies Research Institute, University of Tasmania, Liverpool Street, Hobart, 7000, Australia

## Abstract

**Background:**

Whilst patellofemoral pain is one of the most common musculoskeletal disorders presenting to orthopaedic clinics, sports clinics, and general practices, factors contributing to its development in the absence of a defined arthropathy, such as osteoarthritis (OA), are unclear.

The aim of this cross-sectional study was to describe the relationships between parameters of patellofemoral geometry (patella inclination, sulcus angle and patella height) and knee pain and patella cartilage volume.

**Methods:**

240 community-based adults aged 25-60 years were recruited to take part in a study of obesity and musculoskeletal health. Magnetic resonance imaging (MRI) of the dominant knee was used to determine the lateral condyle-patella angle, sulcus angle, and Insall-Salvati ratio, as well as patella cartilage and bone volumes. Pain was assessed by the Western Ontario and McMaster University Osteoarthritis Index (WOMAC) VA pain subscale.

**Results:**

Increased lateral condyle-patella angle (increased medial patella inclination) was associated with a reduction in WOMAC pain score (Regression coefficient -1.57, 95% CI -3.05, -0.09) and increased medial patella cartilage volume (Regression coefficient 51.38 mm^3^, 95% CI 1.68, 101.08 mm^3^). Higher riding patella as indicated by increased Insall-Salvati ratio was associated with decreased medial patella cartilage volume (Regression coefficient -3187 mm^3^, 95% CI -5510, -864 mm^3^). There was a trend for increased lateral patella cartilage volume associated with increased (shallower) sulcus angle (Regression coefficient 43.27 mm^3^, 95% CI -2.43, 88.98 mm^3^).

**Conclusion:**

These results suggest both symptomatic and structural benefits associated with a more medially inclined patella while a high-riding patella may be detrimental to patella cartilage. This provides additional theoretical support for the current use of corrective strategies for patella malalignment that are aimed at medial patella translation, although longitudinal studies will be needed to further substantiate this.

## Background

Patellofemoral pain is one of the most common musculoskeletal disorders presenting to orthopaedic and sports clinics, as well as general practices [1-5]. Factors contributing to the development of patellofemoral pain in the absence of a defined arthropathy, such as osteoarthritis (OA), are unclear and have predominantly been classified as idiopathic [6].

It has been widely hypothesised that patellofemoral pain syndrome is the result of patellofemoral maltracking and abnormal patella alignment and congruity [7,8]. One study hypothesised that the decrease in contact area is a potential source of pain due to the increased cartilage stress [9]. Previous research has predominantly been descriptive, comparing parameters of patellofemoral geometry in those with and without patellofemoral pain [9-11]. In contrast, studies that have examined the relationship between patellofemoral geometry on pain and cartilage are scarce. The small number of studies that have been performed have shown that greater lateral patella inclination, a shallower femoral sulcus and patella alta (a high-riding patella) are characteristic of subjects with patellofemoral pain [8,9,11-13]. Similarly, a shallow femoral sulcus is thought to reduce the congruency of the patellofemoral joint, and contribute toward instability, being associated with subluxation/dislocation syndromes [14,15]. Nonetheless, the relationship between these indices of patella alignment and congruity and anterior knee pain has not been widely examined. Moreover, it is unclear how these indices may influence patellofemoral joint structures, such as cartilage. Understanding how patellofemoral geometry may be related to joint structure and symptomatology is important, since conservative treatment options such as patellofemoral taping and bracing may help to reduce symptoms and radiological signs of disease, and may ultimately contribute to a reduced incidence of clinical pathology.

The aim of this study was to determine the relationship between indices of patellofemoral geometry and both knee pain and patella cartilage volume.

## Methods

### Study population

Men and women aged 25-60 years were recruited to take part in a study of the relationship between obesity and musculoskeletal diseases by advertising in the local press, at the hospitals in the waiting rooms of private weight loss/obesity clinics, and through community weight loss organisations in order to recruit subjects across the spectrum from normal weight to obese. Subjects were excluded if there was a history of any arthropathy diagnosed by a medical practitioner, prior surgical intervention to the knee including arthroscopy, previous significant knee injury requiring non-weight bearing therapy or requiring prescribed analgesia, malignancy or contraindication to MRI. 250 subjects were recruited and had MRI. Ten subjects were excluded from the study as the quality of their MRI was not interpretable. The study was approved by Alfred Hospital Human Research and Ethics committee (HREC) and the Monash standing research ethics committee. All participants gave informed consent.

### Data collection

Study participants completed a questionnaire that included information on their demographics. Weight was measured to the nearest 0.1 kg (shoes, socks, and bulky clothing removed) using a single pair of electronic scales. Height was measured to the nearest 0.1 cm (shoes and socks removed) using a stadiometer. From these data, BMI was calculated. Pain was assessed by the Western Ontario and McMaster University Osteoarthritis Index (WOMAC) pain subscale, analysed using 100 mm visual analogue scales (VAS) [16]. WOMAC pain subscale consists of 5 items (walking on flat surface, going up/down stairs, lying in bed at night, sitting/lying, and standing upright). Patients were required to answer each question using a 100 mm VAS where 0 = no pain and 100 = extreme pain, producing a range of possible scores of 0-500. A decrease in 1 unit WOMAC pain score indicates a decrease of 1 out of 500 units in amount of pain experienced while performing any of the 5 items.

### Magnetic Resonance Imaging (MRI)

An MRI of the dominant (defined by the lower-extremity a subject used to step off from when initiating gait) knee of each subject was performed [17]. We did not want to bias our results by using both legs as this would have increased the cost of the study without improving the ability to answer the study question, given that there is a clustering effect where 2 knees are used [18]. Knees were imaged in full extension, with muscles relaxed, in the sagittal plane on a 1.5-T whole body magnetic resonance unit (Philips, Medical Systems, Eindhoven, the Netherlands) using a commercial transmit-receive extremity coil. The following sequence and parameters were used: 1) T1-weighted fat saturation 3D gradient recall acquisition in the steady state (58 ms/12 ms/55°, repetition time/echo time/flip angle) with a 16 cm field of view, 60 partitions, 512 × 512 matrix and acquisition time 11 min 56 sec (one acquisition). Axial images were converted from sagittal images, with a matrix of 0.312 mm*0.312 mm.

**Figure 1:**
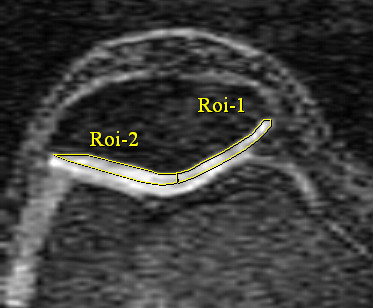
**Medial and lateral patella cartilage volumes were determined by the software Osiris (University of Geneva, Switzerland)**. The patella ridge was used to divide the medial and lateral facets, which were then measured separately on each MRI by manually drawing disarticulation contours around each cartilage boundaries.

Patella cartilage volume was determined by image processing on an independent workstation using the Osiris software (University of Geneva, Switzerland). Patella cartilage volume was isolated from the total volume by manually drawing disarticulation contours around the cartilage boundaries on each section (Figure 1). Medial and lateral patella facet cartilage volumes were further measured separately on each MRI by manually drawing disarticulation contours around the cartilage boundaries on each section as previously described [19]. The coefficient of variation (CV) for the measure was 2.6% [20]. Patella bone volume was determined by drawing contours around the patella boundaries in images 1.5 mm apart on sagittal views in a similar fashion to that described for cartilage volume. The CV for patella bone volume measures was 2.2% [20].

**Figure 2:**
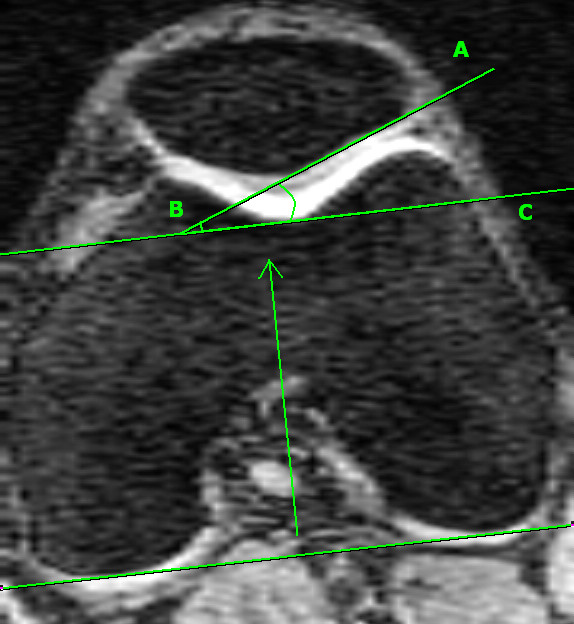
**Lateral condyle-patella angle, measured as the angle between the bony posterior femoral condyles (BC) and the bony lateral patella facet (AB)**.

**Figure 3:**
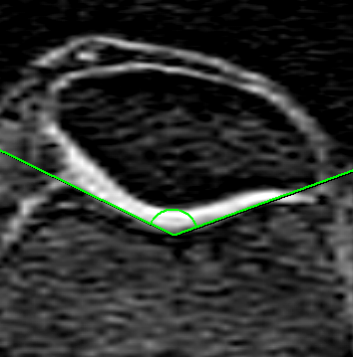
**Sulcus angle defined as the angle formed between lines joining the highest points of the bony medial and lateral condyles and the lowest bony point of the intercondylar sulcus**.

The lateral condyle-patella angle was measured as the angle between the posterior femoral condyles and the lateral inferior bony margin of the patella (Figure 2). An increase in this angle demonstrated more medial patella inclination, whereas a decrease in this angle demonstrated more lateral patella inclination. The femoral sulcus angle was defined by lines joining the highest points of the medial and lateral femoral condyles and the lowest point of the intercondylar sulcus (Figure 3). An increase in the femoral sulcus angle corresponded to a shallower articular surface whereas a decrease in this angle corresponded to a deeper articular surface. Both the lateral condyle-patella angle and femoral sulcus angle were measured at mid-patella level [21], determined by counting all the slices that go through the patella and using the middle slice. In the case when there is an even number of slices, the two middle slices are measured and averaged. The Intraclass Correlation Coefficient (ICC) was 0.98 for both angles. All angles were measured in degrees.

**Figure 4:**
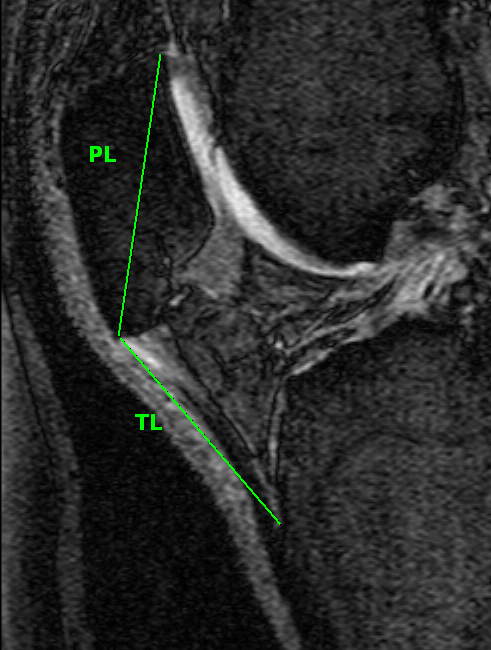
**Insall-Salvati ratio calculated as a ratio of tendon length (BC): patella length (AB) **[22].

Patella height was measured using a method previously described by Insall-Salvati [22] which relates to the length of the patella and the patella tendon (Figure 4). A ratio > 1.2 corresponded to a high-riding patella (patella alta) whereas a ratio < 0.8 was low-riding (patella baja). Ratios between these values were defined as normal. Measures were determined from the sagittal plane at the mid-point of the patella [23], determined by counting the number of slices that go through the patella and using the middle slice, as described above. The ICC was 0.86.

### Statistical Analysis

All outcome variables were initially assessed for normality. Linear regression was used to explore the relationship between parameters of patellofemoral geometry (patella inclination, sulcus angle and patella height) and WOMAC pain score and patella cartilage volume. The multivariate analyses were adjusted for age, gender, BMI, patella cartilage volume and bone size. Adjusting for patella cartilage volume and bone size allowed us to adjust for the structural state of the patellofemoral joint, since this correlates with the radiological grade of patellofemoral OA [24]. The independent samples z-test was used to compare the relationship between parameters of patellofemoral geometry and WOMAC pain and patella cartilage volume in obese and non-obese subjects. A p-value of less than 0.05 (two-tailed) was regarded as statistically significant. All analyses were performed using the SPSS statistical package (standard version 15.0, SPSS, Chicago, IL, USA).

## Results

The demographics of the study cohort are described in Table 1. The mean age of our predominantly female cohort (73.8%) was 45.7 (Standard Deviation (SD) ± 9.4) years, while the mean BMI was of 33.9 kg m^-2 ^(SD ± 9.6) (Table 1). Of the 240 subjects in this study, lateral condyle-patella angle, sulcus angle and Insall-Salvati ratio were only able to be measured in 233, 226, and 222 subjects respectively. This was due to poor quality of the MRI images, which did not allow visualisation and accurate assessment of the required measures. When comparing those who did and did not have interpretable MRI, the only significant difference found was a higher BMI in the uninterpretable group (39.1 ± 6.9 versus 33.9 ± 9.6 kg m^-2^). The study subjects had a mean lateral condyle-patella angle of 19.0 (SD ± 7.0), a mean sulcus angle of 150.8 (SD ± 9.7), and a mean Insall-Salvati Ratio of 1.0 (SD ± 0.1) (Table 1).

**Table 1:** Demographic characteristics of the studied population

	N = 240
Age (years)	45.7 (9.4)
Gender: n (% female)	177 (73.8)
Weight (kg)	94.3 (27.1)
Height (m)	1.7 (0.1)
BMI (kg m^-2^)	33.9 (9.6)
WOMAC pain score	53.9 (82.7)
Patella bone volume (mm^3^)	10966 (2580)
Patella cartilage volume (mm^3^)	2213 (544)
LCPA (degrees) *	19.0 (7.0)
SA (degrees) †	150.8 (9.7)
I-S Ratio‡	1.0 (0.1)

Presented as mean (SD) unless otherwise stated; BMI = Body Mass Index, WOMAC = Western Ontario and McMaster University Osteoarthritis Index, LCPA = lateral condyle-patella angle, SA = sulcus angle, I-S Ratio = Insall-Salvati Ratio

* measured in 233 subjects

† measured in 226 subjects

‡ measured in 222 subjects

Increased lateral condyle-patella angle was negatively associated with WOMAC pain score. In the multivariate analyses, adjusted for age, gender, BMI, patella cartilage volume and bone size as a marker of the state of the joint, for every one degree increase in lateral condyle-patella angle, WOMAC pain score was decreased by 1.57 units (95% CI -3.05, -0.09) (Table 2). We further examined whether this association differed between the obese (BMI ≥ 30) and non-obese (BMI < 30) subjects. There was a dose modification due to obesity in the relationship between lateral condyle-patella angle and WOMAC pain (p = 0.01 for difference between subgroups). For every one degree increase in lateral condyle-patella angle, WOMAC pain was reduced by 3.13 units (95% CI -5.60, -0.67) in the obese, and increased by 0.56 units (95% CI -0.63, 1.75) in the non-obese, although this was not statistically significant (Table 4). There was no significant association between sulcus angle or Insall-Salvati ratio and WOMAC pain score, and no dose modification due to obesity.

**Table 2:** Relationship between patella inclination, sulcus angle and patella height and Western Ontario and McMaster University Osteoarthritis Index (WOMAC) pain score

	Univariate Analysis		Multivariate Analysis*	
	Regression coefficient (95% CI)	P value	Regression coefficient (95% CI)	P value
LCPA	-1.74 (-3.27, -0.21)	0.03	-1.57 (-3.05, -0.09)	0.04
SA	0.25 (-0.85, 1.34)	0.66	0.04 (-1.02, 1.09)	0.95
I-S ratio	57.26 (-13.23, 127.74)	0.11	33.01 (-36.83, 102.85)	0.35

LCPA = lateral condyle-patella angle, SA = sulcus angle, I-S Ratio = Insall-Salvati Ratio, CI = Confidence Interval

*adjusted for age, gender, BMI, patella cartilage volume and bone size

In the univariate analysis, there was no significant association between any of the parameters of patellofemoral geometry measured and medial or lateral patella cartilage volume. After adjusting for age, gender, BMI, patella cartilage volume and bone size, there was a positive association between lateral condyle-patella angle and medial cartilage volume, and a negative association between Insall-Salvati ratio and cartilage volume in the medial compartment (Table 3). For every one degree increase in lateral condyle-patella angle, there was an associated 51.38 mm^3 ^(95% CI 1.68, 101.08 mm^3^) increase in medial patella cartilage volume, while for every one unit increase in Insall-Salvati ratio, there was a 3187 mm^3 ^(95% CI -5510, -864 mm^3^) reduction in medial patella cartilage volume. No similar results were found in the lateral compartment, although there was a trend for a positive association between sulcus angle and lateral patella cartilage volume. When we examined the obese and non-obese subjects separately, there was no dose modification due to obesity in the relationship between lateral condyle-patella angle and Insall-Salvati ratio and patella cartilage volume. However, when we examined the relationship between sulcus angle and medial patella cartilage, there was a stronger effect in the non-obese (regression coefficient -55.61, 95% CI -104.79, -6.43) compared to the obese population (regression coefficient 12.01, 95% CI -35.76, 59.77) (p = 0.05 for difference between subgroups). There was no significant association between sulcus angle and lateral patella cartilage in either the non-obese (regression coefficient 31.12, 95% CI -44.23, 106.48) or the obese subgroup (regression coefficient 53.63, 95% CI -6.93, 114.19) and no dose modification due to obesity (Table 4).

**Table 3:** Relationship between patella inclination, sulcus angle and patella height and patella cartilage volume

	Univariate Analysis		Multivariate Analysis*	
	Regression coefficient (95% CI)	P value	Regression coefficient (95% CI)	P value
**Medial facet**				
LCPA	40.34 (-18.30, 98.98)	0.18	67.23 (22.10, 112.36)	0.004
SA	-23.83 (-66.34, 18.68)	0.27	-23.63 (-56.89, 9.63)	0.16
I-S ratio	-3213.15 (-6140.70, 285.60)	0.03	-3186.89 (-5510.01, -863.77)	0.01
**Lateral facet**				
LCPA	16.90 (-66.20, 100.01)	0.69	48.88 (-12.34, 110.11)	0.12
SA	41.08 (-20.33, 102.49)	0.19	43.27 (-2.43, 88.98)	0.06
I-S ratio	-3390.01 (-7598.65, 818.63)	0.11	-2826.33 (-5985.73, 333.07)	0.08

LCPA = lateral condyle-patella angle, SA = sulcus angle, I-S Ratio = Insall-Salvati Ratio, CI = Confidence Interval

*adjusted for age, gender, BMI, patella cartilage volume and bone size

**Table 4:** Relationship between parameters of patellofemoral geometry and WOMAC pain and patella cartilage volume: the difference between obese and non-obese subjects

	Obese subgroup		Non-obese subgroup		
	Regression coefficient (95% CI)*	P value	Regression coefficient (95% CI)*	P value	P value for difference†
**WOMAC pain score**					
LCPA	-3.13 (-5.60, -0.67)	0.01	0.56 (-0.63, 1.75)	0.36	0.01
SA	0.42 (-1.46, 2.29)	0.66	-0.1 (-0.79, 0.77)	0.98	0.68
I-S ratio	87.80 (-30.86, 206.46)	0.15	-37.58 (-101.45, 26.29)	0.25	0.06
**Medial patella cartilage volume**					
LCPA	86.36 (26.15, 146.58)	0.01	47.81 (-22.37, 117.99)	0.18	0.41
SA	12.01 (-35.76, 59.77)	0.62	-55.61 (-104.79, -6.43)	0.03	0.05
I-S ratio	-3093.84 (-6260.66, 72.98)	0.06	-2943.85 (-6497.61, 603.91)	0.10	0.95
**Lateral patella cartilage volume**					
LCPA	49.36 (-29.20, 127.93)	0.22	48.77 (-52.06, 149.60)	0.34	0.99
SA	53.63 (-6.93, 114.19)	0.08	31.12 (-44.23, 106.48)	0.41	0.64
I-S ratio	-3594.17 (-75463.31, 357.98)	0.07	-2433.12 (-7752.83, 2886.59)	0.37	0.73

LCPA = lateral condyle-patella angle, SA = sulcus angle, I-S Ratio = Insall-Salvati Ratio, CI = Confidence Interval

*adjusted for age, gender, BMI, patella cartilage volume and bone size

†p value calculated using independent samples z-test for difference between subgroups

## Discussion

In a community-based population of adults, an increased lateral condyle-patella angle (a more medially inclined patella) was associated with reduced WOMAC pain score and increased medial patella cartilage volume. In contrast, an increased Insall-Salvati ratio (towards patella alta) was associated with reduced medial patella cartilage volume. These results suggest that whereas both symptomatic and structural benefits occur with medial patella inclination, patella alta is associated with aberrations in patella cartilage morphology.

Although the aetiology of patella pain remains unclear, patellofemoral maltracking is thought to play a central role in the genesis of anterior knee pain [7,25]. Previously, it was shown that women with patellofemoral pain had significantly greater lateral patella inclination compared to healthy controls [11]. Similarly, in the current study, we demonstrated that the lateral condyle-patella angle, which assesses the inclination of the patella relative to the orientation of the femur, was associated with knee pain. In particular, a more medially inclined patella (an increased lateral condyle-patella angle) (see figure 2) was associated with reduced WOMAC knee pain scores, and this was more pronounced in the obese subjects. We also demonstrated that a more medially inclined patella was associated with increased medial patella cartilage volume. Consistent with our findings, a recent MRI study examining people with knee OA demonstrated that a medially oriented patella (largest lateral condyle-patella angle) was associated with the least reduction in lateral patella cartilage [26]. In contrast, a recent radiographic study found that a more laterally inclined patella (an increase in the lateral patella tilt angle) was associated with an increase in the medial patellofemoral joint space [12]. This was regarded by the authors as protective of medial joint space narrowing progression, thereby presuming it to be advantageous to the patellofemoral joint. However, when the patella is laterally inclined, the radiographic patellofemoral joint space will intuitively be widened medially, without necessarily being associated with increased cartilage volume.

In patellofemoral subluxation, dislocation, and pain syndromes, the precipitating event is generally considered to be generated by excessive lateral translation of the patella [27,28]. Conservative treatment for these pathologies has therefore been aimed at medial patella translation via strategies including braces, taping and vastus medialis strengthening [27,29-31]. Likewise, surgical approaches such as medial patellofemoral ligament reconstruction [32-34] can be used to optimise patellar tracking, particularly in patellofemoral instability. Although our findings support an advantage imparted by medial patella inclination, the biomechanical mechanism by which the symptomatic and structural benefits are linked remains unclear. One plausible explanation is that medial inclination of the patella offsets the natural tendency of the patella to track laterally which causes excessive shearing across cartilage. However, since cartilage is not innervated, how pain may arise is even less clear.

We also measured other indices of patellofemoral articulation and alignment. The femoral sulcus angle (see figure 3) measures the depth of the femoral articular surface at the patellofemoral joint, and unlike the lateral condyle-patella angle, is viewed as a measure of joint congruity rather than patella tracking [14,35]. An increased sulcus angle is commonly associated with patella subluxation and dislocation [36,37], and in its most severe form, represents trochlear dysplasia [38]. Nonetheless, our study demonstrated a trend towards a positive association between the femoral sulcus angle and lateral patella cartilage volume. Although it is generally accepted that a shallower sulcus angle is associated with decreased patellofemoral congruency and stability [14,15], and adverse cartilage outcomes [12,26,39], a study in an osteoarthritic population demonstrated a positive association between sulcus angle and patella cartilage volume [19]. Mechanistically, it may be that a shallower sulcus enables a greater articular surface area, which subsequently reduces patellofemoral contact pressures, thus benefiting articular cartilage [19]. However, when analysed separately, there was a negative association between sulcus angle and medial patella cartilage volume in the non-obese population, which was significantly different to the obese population. One hypothesis is that those in the non-obese population are more physically active than those who are obese, therefore it may be that the negative association between sulcus angle and medial patella cartilage in the non-obese subgroup is mediated by physical activity. Those with a shallower sulcus are likely to have greater instability, and may therefore be more prone to cartilage loss during vigorous physical activity. In contrast, we have demonstrated that a high-riding patella is associated with adverse cartilage changes. Previously, patella alta has been shown to lead to higher cartilage loss [26]. The Insall-Salvati ratio is a measure of patella height, calculated as a ratio between the patella tendon and patella length (see figure 4). This ratio helps to identify excessive proximal (patella alta - defined as an Insall-Salvati ratio > 1.2) or distal (patella baja/patella inferra - defined as an Insall-Salvati ratio < 0.8) alignment of the patella relative to the trochlear groove [40]. Both patella alta and baja can alter the biomechanics of the knee joint and have been shown to lead to deleterious structural changes [13,41]. Mechanistically, a higher vertical positioning of the patella (patella alta) corresponds with the largest patellofemoral contact pressures when averaged over the whole range of movement [42], which may structurally manifest as reduced cartilage volume over time.

Our study has several limitations. WOMAC knee pain scores do not differentiate between tibiofemoral or patellofemoral pain. Whilst the Kujala score may be used to assess patellofemoral disorders [43], this data was not available in our study, thus we used the WOMAC knee pain score, which has been widely utilised in previous studies of patellofemoral diseases [44]. Although we did not have radiographic assessment in this study, we adjusted for patella cartilage volume (adjusted for bone size) as we have previously shown that this is strongly related to radiographic grade of OA [24] and is a very sensitive method for detecting early changes of knee OA [45]. Our study was based on MRI which has the benefit of *in vivo *assessment of cartilage and bone. Additionally, our cohort predominantly comprised females, which may have affected our results as patellofemoral joint biomechanics has been shown to differ between males and females [46]. We excluded subjects if there was a history of any arthropathy diagnosed by a medical practitioner, prior surgical intervention, and previous significant knee injury, thus it is possible that some subjects with clinically severe patellofemoral pain syndrome were excluded from our study. Finally, our subjects, who ranged from normal weight to obese, were recruited to take part in a study of the relationship between obesity and musculoskeletal disease. As the BMI of the population was normally distributed, we analysed the population as a whole, however we did perform the independent samples z-test to examine for any difference between the obese and non-obese subjects. Whilst it is possible that those with a high BMI were more likely to have knee pain [47,48], our multivariate analyses were adjusted for BMI. A small number of images were excluded from this study because of their poor quality which tended to occur in those with higher BMI, thus limiting the generalisability of our results to those of vey high BMI. The inability of current imaging equipments to produce the desired image quality in obese subjects is a limitation to today's imaging techniques [49].

## Conclusions

In this study, we have demonstrated that an increased lateral condyle-patella angle (a more medially inclined patella) is associated with decreased WOMAC pain score in a community-based population. Moreover, increased lateral condyle-patella angle and increased sulcus angle are associated with increased medial and lateral patella cartilage respectively, whilst patella alta was associated with reduced medial patella cartilage volume. These results support symptomatic and structural benefits being associated with a medially inclined patella and a shallower sulcus angle, and further support a high-riding patella as a disadvantageous feature of patellofemoral joint biomechanics.

## Abbreviations

OA: Osteoarthritis; MRI: Magnetic Resonance Imaging; WOMAC: Western Ontario and McMaster University Osteoarthritis Index; VAS: Visual Analogue Scale; CV: Coefficient of Variation; ICC: Intraclass Correlation Coefficient; SD: Standard Deviation; BMI: Body Mass Index.

## Competing interests

The authors declare that they have no competing interests.

## Authors' contributions

SKT was involved in data collection, analyses and manuscript preparation; AJT was involved in manuscript preparation; AEW, DMU and GJ were involved in manuscript revision; YW and MD-T were involved in data collection; FMC was involved in manuscript preparation. All authors read and approved the final manuscript.

## Pre-publication history

The pre-publication history for this paper can be accessed here:

http://www.biomedcentral.com/1471-2474/11/87/prepub

## References

[B1] Ireland ML Willson JD Ballantyne BT Davis IM Hip strength in females with and without patellofemoral pain J Orthop Sports Phys Ther 2003 33 11 671 676 14669962 10.2519/jospt.2003.33.11.671

[B2] van Middelkoop M van Linschoten R Berger MY Koes BW Bierma-Zeinstra SM Knee complaints seen in general practice: active sport participants versus non-sport participants BMC Musculoskelet Disord 2008 9 36 10.1186/1471-2474-9-36 18366679 PMC2278141

[B3] Baquie P Brukner P Injuries presenting to an Australian sports medicine centre: a 12-month study Clin J Sport Med 1997 7 1 28 31 10.1097/00042752-199701000-00006 9117522

[B4] Taunton JE Ryan MB Clement DB McKenzie DC Lloyd-Smith DR Zumbo BD A retrospective case-control analysis of 2002 running injuries British Journal of Sports Medicine 2002 36 2 95 101 10.1136/bjsm.36.2.95 11916889 PMC1724490

[B5] Feller JA Amis AA Andrish JT Arendt EA Erasmus PJ Powers CM Surgical biomechanics of the patellofemoral joint Arthroscopy 2007 23 5 542 553 17478287 10.1016/j.arthro.2007.03.006

[B6] Naslund J Naslund U-B Odenbring S Lundeberg T Comparison of symptoms and clinical findings in subgroups of individuals with patellofemoral pain Physiother Theory Pract 2006 22 3 105 118 10.1080/09593980600724246 16848349

[B7] Grelsamer RP Patellar malalignment.[see comment] J Bone Joint Surg 2000 82-A 11 1639 1650 11097456

[B8] Wittstein JR Bartlett EC Easterbrook J Byrd JC Magnetic resonance imaging evaluation of patellofemoral malalignment.[see comment] Arthroscopy 2006 22 6 643 649 16762703 10.1016/j.arthro.2006.03.005

[B9] Sheehan FT Derasari A Brindle TJ Alter KE Understanding patellofemoral pain with maltracking in the presence of joint laxity: complete 3D in vivo patellofemoral and tibiofemoral kinematics Journal of Orthopaedic Research 2009 27 5 561 570 10.1002/jor.20783 19009601 PMC5537740

[B10] O'Donnell P Johnstone C Watson M McNally E Ostlere S Evaluation of patellar tracking in symptomatic and asymptomatic individuals by magnetic resonance imaging Skeletal Radiol 2005 34 3 130 135 10.1007/s00256-004-0867-6 15517249

[B11] Powers CM Patellar kinematics, part II: the influence of the depth of the trochlear groove in subjects with and without patellofemoral pain Phys Ther 2000 80 10 965 973 11002432

[B12] Hunter DJ Zhang YQ Niu JB Felson DT Kwoh K Newman A Kritchevsky S Harris T Carbone L Nevitt M Patella malalignment, pain and patellofemoral progression: the Health ABC Study Osteoarthritis Cartilage 2007 15 10 1120 1127 10.1016/j.joca.2007.03.020 17502158 PMC2042530

[B13] Ward SR Terk MR Powers CM Patella alta: association with patellofemoral alignment and changes in contact area during weight-bearing.[see comment] J Bone Joint Surg Am 2007 89 8 1749 1755 10.2106/JBJS.F.00508 17671014

[B14] Davies AP Costa ML Shepstone L Glasgow MM Donell S The sulcus angle and malalignment of the extensor mechanism of the knee.[see comment][erratum appears in J Bone Joint Surg Br 2001 Jul;83(5):777 Note: Donnell ST [corrected to Donell S]] J bone Joint Surg Br 2000 82 8 1162 1166 10.1302/0301-620X.82B8.10833 11132279

[B15] Nietosvaara Y The femoral sulcus in children. An ultrasonographic study J Bone Joint Surg Br 1994 76 5 807 809 8083274

[B16] Bellamy N Buchanan WW Goldsmith CH Campbell J Stitt LW Validation study of WOMAC: a health status instrument for measuring clinically important patient relevant outcomes to antirheumatic drug therapy in patients with osteoarthritis of the hip or knee J Rheumatol 1988 15 1833 1840 3068365

[B17] Guymer E Baranyay F Wluka AE Hanna F Bell RJ Davis SR Wang Y Cicuttini FM A study of the prevalence and associations of subchondral bone marrow lesions in the knees of healthy, middle-aged women Osteoarthritis Cartilage 2007 15 12 1437 1442 10.1016/j.joca.2007.04.010 17560134

[B18] Sutton AJ Muir KR Jones AC Two knees or one person: data analysis strategies for paired joints or organs Ann Rheum Dis 1997 56 7 401 402 10.1136/ard.56.7.401 9485999 PMC1752405

[B19] Davies-Tuck M Teichtahl AJ Wluka AE Wang Y Urquhart DM Cui J Cicuttini FM Femoral sulcus angle and increased patella facet cartilage volume in an osteoarthritic population Osteoarthritis Cartilage 2008 16 1 131 135 10.1016/j.joca.2007.08.002 17869546

[B20] Jones G Glisson M Hynes K Cicuttini F Sex and site differences in cartilage development: a possible explanation for variations in knee osteoarthritis in later life Arthritis Rheum 2000 43 11 2543 2549 10.1002/1529-0131(200011)43:11<2543::AID-ANR23>3.0.CO;2-K 11083279

[B21] Muellner T Funovics M Nikolic A Metz V Schabus R Vecsei V Patellar alignment evaluated by MRI Acta Orthopaedica Scandinavica 1998 69 5 489 492 9855230 10.3109/17453679808997784

[B22] Insall J Salvati E Patella position in the normal knee joint Radiology 1971 101 1 101 104 10.1148/101.1.101 5111961

[B23] Shabshin N Schweitzer ME Morrison WB Parker L MRI criteria for patella alta and baja Skeletal Radiol 2004 33 8 445 450 10.1007/s00256-004-0794-6 15221214

[B24] Cicuttini FM Wang YY Forbes A Wluka AE Glisson M Comparison between patella cartilage volume and radiological assessment of the patellofemoral joint Clin Exp Rheumatol 2003 21 3 321 326 12846050

[B25] Grelsamer RP Weinstein CH Gould J Dubey A Patellar tilt: the physical examination correlates with MR imaging Knee 2008 15 1 3 8 10.1016/j.knee.2007.08.010 18023186

[B26] Kalichman L Zhang Y Niu J Goggins J Gale D Felson DT Hunter D The association between patellar alignment and patellofemoral joint osteoarthritis features--an MRI study Rheumatology 2007 46 8 1303 1308 10.1093/rheumatology/kem095 17525117

[B27] Dejour H Walch G Nove-Josserand L Guier C Factors of patellar instability: an anatomic radiographic study Knee Surg Sports Traumatol Arthrosc 1994 2 1 19 26 10.1007/BF01552649 7584171

[B28] Post WR Clinical evaluation of patients with patellofemoral disorders Arthroscopy 1999 15 8 841 851 10.1053/ar.1999.v15.015084 10564862

[B29] Herrington L The effect of corrective taping of the patella on patella position as defined by MRI Research in Sports Medicine 2006 14 3 215 223 10.1080/15438620600854785 16967773

[B30] Hinman RS Crossley KM Patellofemoral joint osteoarthritis: an important subgroup of knee osteoarthritis Rheumatology 2007 46 7 1057 1062 10.1093/rheumatology/kem114 17500072

[B31] Whittingham M Palmer S Macmillan F Effects of taping on pain and function in patellofemoral pain syndrome: a randomized controlled trial J Orthop Sports Phys Ther 2004 34 9 504 510 15493518 10.2519/jospt.2004.34.9.504

[B32] Sallay PI Poggi J Speer KP Garrett WE Acute dislocation of the patella. A correlative pathoanatomic study American Journal of Sports Medicine 1996 24 1 52 60 10.1177/036354659602400110 8638754

[B33] Steiner TM Torga-Spak R Teitge RA Medial patellofemoral ligament reconstruction in patients with lateral patellar instability and trochlear dysplasia American Journal of Sports Medicine 2006 34 8 1254 1261 10.1177/0363546505285584 16567459

[B34] Mikashima Y Kimura M Kobayashi Y Asagumo H Tomatsu T Medial patellofemoral ligament reconstruction for recurrent patellar instability Acta Orthopaedica Belgica 2004 70 6 545 550 15669454

[B35] Hohe J Ateshian G Reiser M Englmeier KH Eckstein F Surface size, curvature analysis, and assessment of knee joint incongruity with MRI in vivo Magn Reson Med 2002 47 3 554 561 10.1002/mrm.10097 11870843

[B36] McNally EG Ostlere SJ Pal C Phillips A Reid H Dodd C Assessment of patellar maltracking using combined static and dynamic MRI Eur Radiol 2000 10 7 1051 1055 10.1007/s003300000358 11003396

[B37] Kujala UM Osterman K Kormano M Nelimarkka O Hurme M Taimela S Patellofemoral relationships in recurrent patellar dislocation J Bone Joint Surg Br 1989 71 5 788 792 2584248 10.1302/0301-620X.71B5.2584248

[B38] Pfirrmann CW Zanetti M Romero J Hodler J Femoral trochlear dysplasia: MR findings Radiology 2000 216 3 858 864 10966723 10.1148/radiology.216.3.r00se38858

[B39] Kalichman L Zhang Y Niu J Goggins J Gale D Zhu Y Felson DT Hunter DJ The association between patellar alignment on magnetic resonance imaging and radiographic manifestations of knee osteoarthritis Arthritis research & therapy 2007 9 2 R26 10.1186/ar2138 PMC1906802 17343731

[B40] Brattstrom H Patella alta in non-dislocating knee joints Acta Orthop Scand 1970 41 5 578 588 5507904 10.3109/17453677008991549

[B41] Noyes FR Wojtys EM Marshall MT The early diagnosis and treatment of developmental patella infera syndrome Clin Orthop Relat Res 1991 265 241 252 2009665

[B42] Luyckx T Didden K Vandenneucker H Labey L Innocenti B Bellemans J Is there a biomechanical explanation for anterior knee pain in patients with patella alta?: influence of patellar height on patellofemoral contact force, contact area and contact pressure J Bone Joint Surg Br 2009 91 3 344 350 10.1302/0301-620X.91B3.21592 19258610

[B43] Kujala UM Jaakkola LH Koskinen SK Taimela S Hurme M Nelimarkka O Scoring of patellofemoral disorders Arthroscopy: The Journal of Arthroscopic & Related Surgery 1993 9 2 159 163 10.1016/s0749-8063(05)80366-4 8461073

[B44] Hinman RS Crossley KM McConnell J Bennell KL Efficacy of knee tape in the management of osteoarthritis of the knee: blinded randomised controlled trial BMJ 2003 327 7407 135 10.1136/bmj.327.7407.135 PMC165705 12869456

[B45] Cicuttini FM Wluka AE Hankin J Stuckey S Comparison of patella cartilage volume and radiography in the assessment of longitudinal joint change at the patellofemoral joint J Rheumatol 2004 31 7 1369 1372 15229959

[B46] Csintalan RP Schulz MM Woo J McMahon PJ Lee TQ Csintalan RP Schulz MM Woo J McMahon PJ Lee TQ Gender differences in patellofemoral joint biomechanics Clin Orthop Relat Res 2002 402 260 269 10.1097/00003086-200209000-00026 12218492

[B47] Adamson J Ebrahim S Dieppe P Hunt K Prevalence and risk factors for joint pain among men and women in the West of Scotland Twenty-07 study Annals of the Rheumatic Diseases 2006 65 4 520 524 10.1136/ard.2005.037317 16126799 PMC1798081

[B48] Jinks C Jordan KP Blagojevic M Croft P Predictors of onset and progression of knee pain in adults living in the community. A prospective study Rheumatology 2008 47 3 368 374 10.1093/rheumatology/kem374 18263594

[B49] Uppot RN Sahani DV Hahn PF Gervais D Mueller PR Impact of Obesity on Medical Imaging and Image-Guided Intervention Am J Roentgenol 2007 188 2 433 440 10.2214/AJR.06.0409 17242253

